# Perspectives of the European Society of Endocrinology (ESE) and the European Society of Paediatric Endocrinology (ESPE) on rare endocrine disease

**DOI:** 10.1007/s12020-021-02652-x

**Published:** 2021-03-19

**Authors:** Martin Reincke, Anita Hokken-Koelega

**Affiliations:** 1 President-elect, European Society of Endocrinology, Medizinische Klinik und Poliklinik IV, Klinikum der Universität München, LMU München, München, Germany; 2 Secretary General, European Society for Paediatric Endocrinology (ESPE), Erasmus University Medical Center, Rotterdam, The Netherlands

**Keywords:** Endo-ERN, Rare disease, Patient perspective, Endocrinology

## Abstract

**Purpose:**

Rare diseases affect <1 in 2000 people. Despite their rarity, they collectively affect ~30 million people across Europe. The aim of this article is to present the view of our European endocrine societies on the care of patients with rare endocrine conditions.

**Methods:**

We evaluated the current situation of patients with rare endocrine disease and present the joint views of the European Society for Endocrinology (ESE) and the European Society for Pediatric Endocrinology (ESPE) on how the endocrine disciplines can support and contribute to a better health of patients with rare endocrine conditions in Europe.

**Results:**

Rare diseases pose many challenges, including early diagnosis and innovative treatment options. Rare endocrine diseases can be found among inherited disorders, cancers, and conditions associated with metabolic disorders such as diabetes, calcium and bone metabolism, lipid metabolism, hypogonadism, and adrenal, pituitary, and thyroid dysfunction. According to the European Registries for Rare Endocrine conditions, there are over 440 distinct rare diseases that affect the endocrine system. Rare endocrine diseases are often chronic and life-threatening.

**Conclusions:**

ESE and ESPE support a strategic plan to address unmet needs in the area of rare endocrine conditions. The EU should continue to evolve and expand its plans for funding European Reference Networks so that they can expand their activities.

## Introduction: what we stand for!

The European Society of Endocrinology (ESE) was founded in 2006 and has currently 4.537 members. The European Society for Paediatric Endocrinology (ESPE) was founded in 1965 and has 1.347 members. In association with the national endocrine societies we represent around 20,000 endocrinologists and 7000 pediatric endocrinologists in Europe, as well as millions of patients with endocrine diseases. Together, our two societies cover endocrine health and disease across the lifespan, from birth over childhood, adolescence, adulthood and ageing.

In this article, the community of European endocrine specialists presents its views on how, based on the science we develop, the clinical work we do and our health-related expectations towards the future, the endocrine discipline can support and contribute to a better health of patients with rare endocrine conditions in Europe.

## Rare endocrine disease in Europe: a challenge

Rare diseases, which affect <1 in 2000 people [1], are less well known and researched, but cover a wide range of conditions. Despite their rarity, these conditions collectively affect ~30 million people across Europe [2] and come with particular challenges related to time to diagnosis, investment into research and access to innovative treatment options. Due to the limited amount of strong epidemiological data, the real prevalence and incidence of many of these rare diseases is not known.

Rare endocrine diseases include inherited disorders (e.g., congenital adrenal hyperplasia), cancer (e.g., multiple endocrine neoplasia syndromes), and conditions associated with metabolic disorders such as diabetes, calcium and bone metabolism, lipid metabolism, hypogonadism, and adrenal, pituitary, and thyroid dysfunction. According to the European Registries for Rare Endocrine [3] conditions there are over 440 distinct rare diseases that affect the endocrine system [4].

It is estimated that it takes on average 4 years to receive a rare disease diagnosis [5]. Rare endocrine diseases are often chronic and life-threatening and accurate estimations of their genuine economic costs and societal burden have not been properly quantified in the EU. Many rare diseases involve pediatric populations with severe implications for societal and health care costs, and limited future opportunities in life for the individuals affected by these diseases and their families. New strategies and incentives to fund research and clinical trials for conditions that affect a small number of patients—and therefore do not represent attractive commercial value for innovative medicines—need to be developed.

## European Reference Networks (ERNs)

ERNs have been instrumental in creating clearly defined structures for sharing knowledge and care coordination for specific rare diseases across the EU. Certain ERNs incorporate rare endocrine diseases into their health remit including ENDO-ERN (The European Reference Network on Rare Endocrine conditions), BOND-ERN (The European Reference Network on Rare Bone Diseases, MetabERN (The European Reference Network for Hereditary Metabolic Diseases), EURACAN (The European Reference Network for Rare Adult Solid Cancers), and EuRRECa (The European Registries for Rare Endocrine Conditions). These knowledge-based networks have been critical in shaping the rare disease policy space, however, greater support from the EU is needed to improve their functionality and to improve health outcomes in rare disease patient populations.

Although Europe is ahead of other regions regarding reference networks, the level of funding is not sufficient to allow for lead research projects to be undertaken within these networks. Funding and resources to manage projects and ERN infrastructure and to be able to collect good quality data in these registries is required, as currently endocrinologists focused in this area do not have the capacity to be involved with these programs at large.

Experts report that many countries in Europe are not aware of the existence of these reference networks, and advise that increased promotion within the medical community and towards European and national institutions is needed.

The EU should continue evolving and expanding funding for ERNs so that they can expand their activities, for example through the research program Horizon Europe, which should play a more prominent role in supporting the ERNs due to their significance for European research in rare paediatric endocrine diseases, rare endocrine cancers and other rare diseases. Increased funding would also help the ERNs better coordinate the projects and their infrastructure, and compensate patients and the medical/academic communities for giving their valuable time to the study of these diseases.

Both societies strongly support the ERNs in general and specifically Endo-ERN since their foundation. The support is intellectually, organizational, financially and by active policy and advocacy. Rare diseases are an important part of the ESE White Paper Statement *Hormones in European Health Policies: How endocrinologists can contribute towards a healthier Europe* [6], which has been endorsed by the ESPE. Both societies have obliged themselves to a strategic plan to address unmet needs in the areas of obesity, endocrine cancer, exposure to endocrine disrupting chemicals and rare endocrine diseases.

Both societies have established a rare disease committee consisting of society members and Endo-ERN representatives. The main aims of the committees are to provide and develop a common platform to foster collaboration, co-operation, and coordination of activities in the area of rare diseases between the societies and Endo-ERN in terms of:

- Educational needs and collaborative solutions
- To write clinical guidelines
- To develop collaborations with other rare disease networks
- To raise awareness of research & clinical trials
- To promote and facilitate cross border care
- To improve awareness and engagement of researchers and clinicians with rare disease registries
- To provide a platform for collaboration with Patient Advocacy Groups
- To raise public awareness of rare diseases

## Rare endocrine diseases and COVID-19

People living with certain rare diseases may have a higher risk of serious illness from COVID-19. There is uncertainty around the risks of serious disease in this community due to the difficulties in prognosis and the small number of people affected, who can often have very individualized symptoms and co-morbidities. Further research in this area and support for some of the most vulnerable patients is needed, whose feelings of isolation may be exacerbated at this time. According to a study by EURORDIS, COVID-19 caused severe disruptions of care for 83% of people living with a rare disease (http://download2.eurordis.org/rbv/covid19survey/covid_infographics_final.pdf).

The ESE Rare Disease Committee, alongside ENDO-ERN, have engaged in an initiative to collect essential data concerning specific groups of patients with rare endocrine conditions, who are also affected by COVID-19. This project is designed to understand the occurrence of a wide range of conditions and allow the clinical networks to objectively map the conditions and activity. ESPE developed a Covid-19 hub on its website (https://www.eurospe.org/patients/espe-covid-19-hub/) for children and adolescents with life threatening rare endocrine disorders, including diabetes mellitus type 1. Similar initiatives should be stimulated across the EU to obtain a better understanding of the links between rare (endocrine) diseases and COVID-19. On a more positive note, COVID-19 has fostered the uptake of digital tools for patients, including for example virtual consultations. These digital tools may help to improve care and patient outcomes in the future.

## Rare endocrine diseases and patient advocacy groups

Finally, both societies have a clear standing in support of patient advocacy groups. Their input into our rare disease activities are paramount for success. Therefore, the last word will have here one of the ERN representatives. Johan de Graaf, ERN patient representative, suffering from a pituitary adenoma and hypopituitarism, states:

*“As a patient, I really appreciate the founding of ERNs because for the first time, patients are really part of a network in which they can work side by side with health care professionals and give their opinion on important matters concerning them. For example, endocrine patients all over Europe were recently asked for their opinion on patient information materials and their unmet needs in medical research. The outcomes of these surveys will be used to shape the research agenda and will give insight into how to reach out to even more patients in the future. Patient representatives are involved on a voluntary basis and though Endo-ERN does its utmost to make their involvement possible, improved funding mechanisms would enable the ERNs to compensate patients better for their time”*.

We, as European societies with close links to our national European societies and international collaborations all over the globe, will work along this line. Moreover, we are convinced that continuous financial and policy support by the organs of the European Union is essential to materialize health benefits for patients with rare endocrine diseases in the near future.
